# Viral DNA Binding Protein SUMOylation Promotes PML Nuclear Body Localization Next to Viral Replication Centers

**DOI:** 10.1128/mBio.00049-20

**Published:** 2020-03-17

**Authors:** Miona Stubbe, Julia Mai, Christina Paulus, Hans Christian Stubbe, Julia Berscheminski, Maryam Karimi, Samuel Hofmann, Elisabeth Weber, Kamyar Hadian, Ron Hay, Peter Groitl, Michael Nevels, Thomas Dobner, Sabrina Schreiner

**Affiliations:** a Institute of Virology, School of Medicine, Technical University Munich, Neuherberg, Germany; b Biomedical Sciences Research Complex, University of St. Andrews, St. Andrews, United Kingdom; c Department of Medicine II, University Hospital, LMU Munich, Munich, Germany; d Heinrich Pette Institute, Leibniz Institute for Experimental Virology, Hamburg, Germany; e Assay Development and Screening Platform, Institute of Molecular Toxicology and Pharmacology, Helmholtz Zentrum München, Neuherberg, Germany; f Wellcome Trust Centre for Gene Regulation and Expression, College of Life Sciences, University of Dundee, Dundee, United Kingdom; g Helmholtz Zentrum München, Institute of Virology, Neuherberg, Germany; Princeton University

**Keywords:** DNA binding protein, E2A/DBP, HAdV, human adenovirus, PML-NB, SUMO, Sp100, virus, replication centers, transcription

## Abstract

Human adenoviruses (HAdVs) have developed mechanisms to manipulate cellular antiviral measures to ensure proper DNA replication, with detailed processes far from being understood. Host cells repress incoming viral genomes through a network of transcriptional regulators that normally control cellular homeostasis. The nuclear domains involved are promyelocytic leukemia protein nuclear bodies (PML-NBs), interferon-inducible, dot-like nuclear structures and hot spots of SUMO posttranslational modification (PTM). In HAdV-infected cells, such SUMO factories are found in close proximity to newly established viral replication centers (RCs) marked by the adenoviral DNA binding protein (DBP) E2A. Here, we show that E2A is a novel target of host SUMOylation, leading to PTMs supporting E2A function in promoting productive infection. Our data show that SUMOylated E2A interacts with PML. Decreasing SUMO-E2A protein levels by generating HAdV variants mutated in the three main SUMO conjugation motifs (SCMs) led to lower numbers of viral RCs and PML-NBs, and these two structures were no longer next to each other. Our data further indicate that SUMOylated E2A binds the host transcription factor Sp100A, promoting HAdV gene expression, and represents the molecular bridge between PML tracks and adjacent viral RCs. Consequently, E2A SCM mutations repressed late viral gene expression and progeny production. These data highlight a novel mechanism used by the virus to benefit from host antiviral responses by exploiting the cellular SUMO conjugation machinery.

## Abstract (precis)

PML nuclear bodies (PML-NBs) are implicated in general antiviral defense based on recruiting host restriction factors; however, it is not understood so far why viruses would establish viral replication centers (RCs) juxtaposed to such “antiviral” compartments. To understand this enigma, we investigate the cross talk between PML-NB components and viral RCs to find the missing link connecting both compartments to promote efficient viral replication and gene expression. Taken together, the current concept is more intricate than originally believed, since viruses apparently take advantage of several specific PML-NB-associated proteins to promote productive infection. Simultaneously, they efficiently inhibit antiviral measures to maintain the viral infectious program. Our data provide evidence that SUMOylation of the viral RC marker protein E2A represents the basis of this virus-host interface and regulates various downstream events to support HAdV productive infection. These results are the basis of our current attempts to generate and screen for specific E2A SUMOylation inhibitors to constitute novel therapeutic approaches to limit and prevent HAdV-mediated diseases and mortality of immunosuppressed patients.

## INTRODUCTION

Promyelocytic leukemia protein nuclear bodies (PML-NBs) are interferon-inducible, matrix-associated multiprotein complexes, appearing as punctate nuclear structures (1). Most mammalian cells contain up to 30 PML-NBs, although their number, function, composition, and size depend on cell type, cell cycle stage, and stress responses (2–5). Various proteins localize in these domains, either constitutively or transiently depending on conditions such as stress, interferon expression, transformation, or viral infection (1, 6). Constitutive factors localized in PML-NBs are the tumor suppressor PML, the transcriptional modulator Sp100, the chromatin remodeling factor Daxx, Bloom syndrome RecQ like-helicase (BLM), and SUMO (7, 8). According to electron microscopy studies, PML-NBs are ringlike structures comprising PML and Sp100 (9, 10). Posttranslational modification (PTM) of PML and PML-associated proteins by the addition of the small ubiquitin-like modifier SUMO regulates PML-NB function, integrity, and formation (6, 10, 11). PML-NBs are known to be hot spots for SUMOylation, with most of the SUMOylation pathway enzymes localized within these structures, and the vast majority of PML-NB-associated proteins are modified by SUMO (6). Intriguingly, SUMO-1 is mainly found in the PML and Sp100 protein shells, while SUMO-2/3 chains are also localized in the interior of PML-NBs (12).

Mammalian cells encode five different isoforms of SUMO: SUMO-1, -2, -3, and -4 and the recently discovered SUMO-5 (13–17). SUMO proteins are covalently conjugated to substrate proteins via a three-step enzymatic pathway and are usually attached to the lysine residues of the substrates, which are part of the short SUMO conjugation motif (SCM) consensus sequence ΨKXE (where Ψ is a large hydrophobic amino acid, generally isoleucine, leucine, or valine; K is the lysine residue that is modified; X is any residue; and E is a glutamic acid) (18–22). Since SUMO PTM regulates an immense number of cellular functions, it is not surprising that different viruses have developed mechanisms enabling them to modulate this process for their benefit (6, 11, 22–30). Besides many functions, PML-NBs are part of a host cell antiviral defense structure and a mediator in PTM and modulation of cellular proteins. DNA viruses such as human adenoviruses (HAdVs), herpesviruses, polyomaviruses, and papillomaviruses localize their genomes next to PML-NBs (31–36). The formation of replication and transcription domains (RCs [replication centers]) often takes place in close proximity to PML-NBs (37). Hence, all these viruses have developed different strategies to counteract PML-NBs’ antiviral activity (32, 38, 39). Nevertheless, the exact molecular mechanism underlying the subcellular localization of RCs at PML-NBs during infection is not fully understood; likely, the viruses benefit from distinct PML-NB components.

The HAdV early protein E4orf3 suppresses PML-NBs’ antiviral activity by reorganizing, and thereby disrupting, them into track-like structures (31). Components of PML-NBs that negatively influence HAdV gene expression are either degraded by the HAdV E3 ubiquitin ligase complex (40–44) or inactivated by displacement into the interior of HAdV RCs away from contact with newly synthesized mRNA occurring at the outer rim of these structures (45). HAdVs have developed the ability to benefit from certain components of PML-NBs but only when these factors are retained in PML track structures in close vicinity to RCs where active viral transcription takes place (30, 45).

Sp100 is one of the main constituents of PML-NBs, with transcription-regulating properties (46–48). Four Sp100 isoforms are expressed in humans, Sp100A, Sp100B, Sp100C, and Sp100HMG (46, 49–53), all of which are covalently modified by SUMO (54). It has been shown that Sp100A leads to increased chromatin decondensation, while the isoforms Sp100B, -C, and -HMG promote chromatin condensation (55). Recently, we reported that during HAdV infection, Sp100A is retained in PML tracks to amplify HAdV gene expression at the transcriptional level. In contrast, Sp100 isoform B is a host restriction factor and counteracted by active displacement into viral RCs (45).

HAdV RCs are marked by the viral protein E2A (56). This early HAdV DNA binding protein (DBP) is essential for efficient viral replication (57–60), regulation of viral gene expression (61), mRNA stability (62, 63), and virion assembly (64). HAdV RCs are located next to PML-NBs, known hot spots for SUMOylation processes (6), which are relocalized into PML tracks by the virus (31–36). SUMOylation affects protein-protein interactions and the subcellular localization of substrates (65), and it is known that PML-NBs interact with SUMOylated proteins. We therefore aimed to investigate whether adenoviral E2A is a potential SUMOylation target during HAdV infection processes and whether this plays a role in the positioning of PML tracks in proximity to viral RCs.

Here, we show that adenoviral E2A is SUMO modified, facilitating novel E2A protein functions and host interactions. We demonstrate that these virus-host interactions lead to the positioning of PML tracks next to viral RCs. Our results reveal a novel role of E2A in recruiting Sp100A and increasing Sp100A protein levels to promote HAdV late gene expression. Sp100A/E2A binding is not dependent on the presence of the PML scaffold protein, suggesting that Sp100A is involved in regulating the localization of PML tracks adjacent to HAdV RCs. Consequently, E2A SUMOylation contributes to a productive HAdV life cycle by exploiting beneficial host components within virus-induced PML tracks, which we hypothesize are the sites of active transcription surrounding the newly formed viral RC in the host cell nucleus.

## RESULTS

### HAdV E2A is a novel target of the host SUMO conjugation machinery.

**FIG 1:**
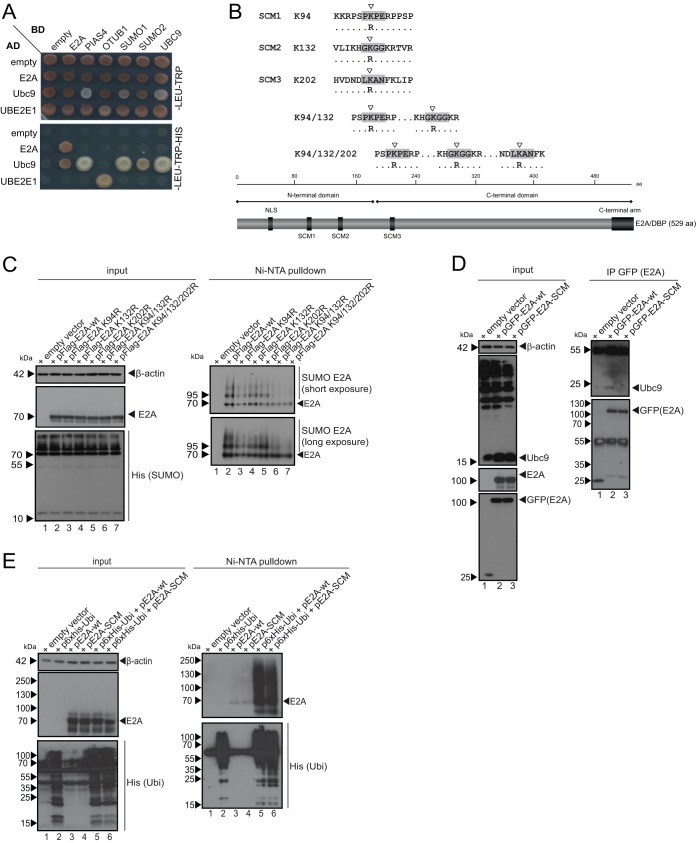
E2A harbors at least three SUMO conjugation motifs. (A) E2A, PIAS4, OTUB1, SUMO-1, SUMO-2, Ubc9, UBE2E1, and empty vector constructs were cotransformed into the yeast strain PJ69-7A as indicated. AD and BD empty vectors were used as controls (empty). Cotransformation was confirmed by plating cells onto agar plates lacking Leu and Trp (containing His). The interaction of candidate proteins was assessed by spotting cells onto agar plates lacking Leu, Trp, and His. (B) Schematic representation of the E2A protein structure with amino acid (aa) numbering above. Known protein domains are indicated by the NLS (nuclear localization signal), N-terminal arm, and C-terminal arm. Below are depicted point mutations that change lysine residues to arginine residues within the SUMO conjugation motifs (SCM). (C) HeLa cells stably expressing 6×His–SUMO-2 were transfected with 5 μg of pCMX3b-Flag E2A (pFlag-E2A-wt) (lane 2) and pCMX3b-Flag E2A (pFlag-E2A) variants containing mutations in SCMs (lanes 3 to 7). Forty-eight hours after transfection, whole-cell lysates were prepared with guanidinium chloride buffer, subjected to Ni-NTA purification of 6×His–SUMO conjugates, and separated by SDS-PAGE prior to immunoblot analyses. Ni-NTA-purified proteins and input levels of cell lysates were detected using mAb B6-8 (anti-E2A), 6×His mAb (anti-6×His tag), and mAb AC-15 (anti-β-actin). Molecular weights in kilodaltons are indicated on the left, and relevant proteins are indicated on the right. (D) H1299 cells were transfected with 5 μg of pGFP-E2A-wt (lane 2) and pGFP-E2A-SCM (lane 3). After 30 h, cells were harvested, and total-cell lysates were prepared. Immunoprecipitation (IP) was performed using polyclonal antibody (pAb) ab290 (anti-GFP), and proteins were subjected to immunoblot analyses. Input levels of total-cell lysates and coprecipitated proteins were detected using mAb B6-8 (anti-E2A), pAb ab290 (anti-GFP), mAb C-12 (anti-Ubc9), and mAb AC-15 (anti-β-actin). Molecular weights in kilodaltons are indicated on the left, and relevant proteins are indicated on the right. (E) H1299 cells were transfected with 10 μg of p6×His-Ubi and 5 μg of pE2A-wt or pE2A-SCM. Thirty hours after transfection, cells were harvested, and whole-cell lysates were prepared with guanidinium chloride buffer. After Ni-NTA purification of 6×His-SUMO conjugates and separation by SDS-PAGE, immunoblot analyses were performed using mAb B6-8 (anti-E2A), 6×His mAb (anti-6×His tag), and mAb AC-15 (anti-β-actin). Molecular weights in kilodaltons are indicated on the left, and relevant proteins are indicated on the right.

HAdVs utilize host SUMOylation processes to enhance viral replication and evade cellular immune responses (66). Here, we searched for potential interactors by yeast two-hybrid (Y2H) experiments and revealed in at least one Y2H combination a novel direct interaction between E2A and the SUMO-conjugating enzyme Ubc9 but not with the SUMO E3 ligase PIAS4 (Fig. 1A). It is a relatively common phenomenon in Y2H assays that one combination reveals more efficient parring than the other. Y2H assays are based on the functional cooperation of the two tags that form a functional transcription factor leading to the detection of an interaction. Here, it can well be that due to a steric hindrance in one combination, the transcription factor is not efficiently formed, resulting in no Y2H signal. To ensure specificity, we confirmed the interaction of Ubc9 with various elements of the SUMO conjugation machinery, such as PIAS4, SUMO-1, and SUMO-2. We verified that there was no interaction of Ubc9 with components of the ubiquitin pathway, such as the DUB (deubiquitinating enzyme) OTUB1 (Fig. 1A), and a UBE2E1 and OTUB1 interaction, used as a positive control, was detected, as expected.

Based on these findings, we used SUMO GPS software (67, 68) to determine putative SCMs (SUMO conjugation motifs) within the E2A protein. E2A SCMs were identified, and the lysines were exchanged for arginines by site-directed mutagenesis (Fig. 1B). E2A SCMs that we identified did not correspond to the usual canonical motif; however, it was previously reported that SUMO can also bind to noncanonical motifs, especially under stress conditions (69, 70). Wild-type E2A (E2A wt) and E2A SCM variants with Flag epitopes were then transfected into human cells stably expressing His–SUMO-2 (Fig. 1C). Ni-nitrilotriacetic acid (NTA)-purified His–SUMO-2 conjugates and total-cell lysates were subjected to Western blot analyses, which revealed an E2A signal at 72 kDa and higher-migrating protein bands (Fig. 1C, right, lane 2). Since covalent SUMO attachment to a substrate protein increases the molecular weight by 20 to 40 kDa, these higher-migrating bands correspond to SUMOylated moieties of the adenoviral protein. We detected less SUMO binding to E2A K94R, K132R, and K202R than to the wt protein (pE2A-wt) (Fig. 1C, right, lanes 2 to 5). pE2A-K94/132R and the triple-SCM mutant pE2A-K94/132/202R (pE2A-SCM), generated to efficiently reduce covalent SUMOylation of the viral factor, showed even less SUMO binding; in particular, E2A SUMO conjugation was drastically diminished when all three putative SCMs were disrupted (Fig. 1C, lane 7), but we did not observe a complete loss of SUMO PTM for the E2A protein.

To further confirm that Ubc9 interacts with E2A in mammalian systems and thereby serves as a SUMO-conjugating enzyme for E2A, we transfected cells with pE2A-wt and pE2A-SCM plasmids tagged with green fluorescent protein (GFP)-expressing epitopes. Immunoprecipitation analyses of GFP-tagged E2A and subsequent staining for endogenous Ubc9 revealed an interaction between E2A and Ubc9 (pGFP-E2A-wt) (Fig. 1D, right, lane 2). As this interaction was absent in cells transfected with the triple-SCM mutant pE2A-K94/132/202R (pGFP-E2A-SCM) (Fig. 1D, right, lane 3), we included this variant in further investigations of the role of E2A SUMO PTM.

Next, we tested whether the lysine-to-arginine exchange in the E2A SCM simultaneously affects E2A ubiquitin PTM or only SUMOylation. Therefore, we transfected E2A wt and E2A SCM versions into human cells in combination with His-ubiquitin (His-Ubi) (Fig. 1E). Ni-NTA-purified His-Ubi conjugates and total-cell lysates were subjected to Western blot analyses and showed similar ubiquitin PTMs above 72 kDa when E2A was detected with a specific antibody (Ab) after His purification (Fig. 1E, right, lanes 5 and 6).

### Generation and validation of an E2A SCM mutant virus.

**FIG 2:**
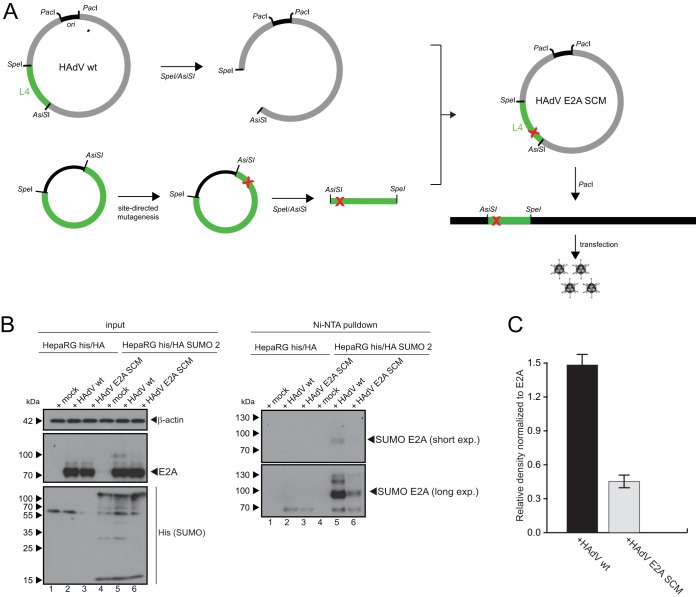
Generation and validation of the E2A SCM mutant virus. (A) Point mutations were inserted into the *e2a* gene in late HAdV region L4 from nucleotides (nt) 21438 to 27081 (pL41513) by site-directed mutagenesis. The L4 plasmid and bacmid pH5*pg*4100 were linearized by AsiSI plus SpeI digestion, and the 5.6-kb AsiSI/SpeI fragment from pH5*pg*4100 was replaced with the corresponding fragment from the mutated pL4 construct. The viral genome was released by PacI digestion and used to transfect H1299 cells, followed by virus propagation and isolation. (B) HepaRG cells stably expressing His/HA or His/HA–SUMO-2 were infected with wt HAdV and the HAdV E2A SCM mutant at a multiplicity of infection of 20 focus-forming units (FFU)/cell. The cells were harvested at 24 h p.i., and whole-cell lysates were prepared with guanidinium chloride buffer, subjected to Ni-NTA purification of 6×His-SUMO conjugates, and separated by SDS-PAGE. Input levels of total-cell lysates and Ni-NTA-purified conjugates were detected with mAb B6-8 (anti-E2A), 6×His mAb (anti-6×His tag), and mAb AC-15 (anti-β-actin). Molecular weights in kilodaltons are indicated on the left, and relevant proteins are indicated on the right. (C) Densitometric analysis of E2A SUMOylation levels in panel B (lanes 5 and 6), quantified with ImageJ (version 1.45s) software, normalized to the respective steady-state E2A levels in HAdV wt- and HAdV E2A SCM-infected cells. The bar plot represents average values and standard deviations calculated based on data from three separate experiments.

To elucidate the role of E2A SUMOylation during the course of adenoviral infection, we generated a mutant virus mimicking the mutant pE2A-SCM construct, where lysine residues K94/132/202 are replaced by arginine to reduce SUMOylation of the viral protein. We used a two-plasmid system with a large viral bacmid and a small region-specific plasmid (L4-Box) to insert point mutations into E2A in the 36-kb linear genome of HAdV (Fig. 2A). Analyses of Ni-NTA-purified samples from infected HepaRG His/HA SUMO-2 cells revealed that there is less formation of E2A SUMO chains during infection with the E2A SCM mutant virus (HAdV E2A SCM) than with wt virus (HAdV wt) infection (Fig. 2B, right, lanes 5 and 6). E2A SUMOylation in E2A SCM mutant-infected cells was reduced by 70% compared to HAdV wt infection (Fig. 2C). These results concurred with data from the transfection experiments described above (Fig. 1C and D), confirming that E2A is a novel viral target of the cellular SUMO conjugation machinery and that the triple-SCM mutation of K94/132/202 efficiently reduced but did not completely abolish PTM.

### E2A SUMOylation controls PML binding to the viral factor in infected cells.

**FIG 3:**
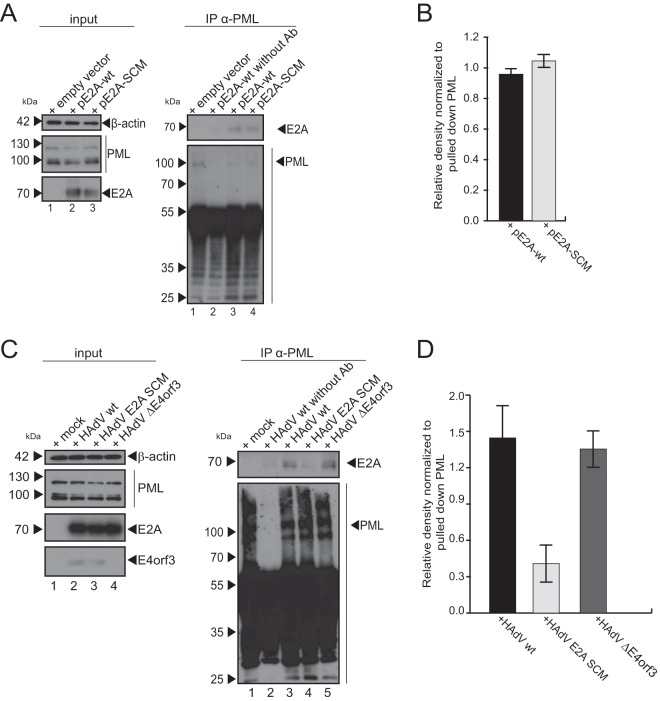
E2A SUMOylation controls E2A/PML interaction during HAdV infection. (A) HepaRG cells transfected with 5 μg of pE2A-wt (left, lane 2; right, lanes 2 and 3) and pE2A-SCM (left, lane 3; right, lane 4) were harvested 30 h after transfection. After preparation of total-cell lysates, immunoprecipitation of PML was performed using a polyclonal rabbit antibody raised against PML protein pAb NB100-59787 (anti-PML), and proteins resolved by SDS-PAGE were visualized by immunoblotting. Input levels of total-cell lysates and coprecipitated proteins were detected using mAb B6-8 (anti-E2A), pAb NB100-59787 (anti-PML), and mAb AC-15 (anti-β-actin). Molecular weights in kilodaltons are indicated on the left, and relevant proteins are indicated on the right. (B) Densitometric analyses of E2A levels immunoprecipitated with PML in panel A (lanes 3 and 4), quantified with ImageJ (version 1.45s) software, normalized to the respective levels of immunoprecipitated PML in pE2A-wt- and pE2A-SCM-transfected cells. The bar plot represents average values and standard deviations calculated based on data obtained in three separate experiments. (C) HepaRG cells were infected with HAdV wt (left, lane 2; right, lane 3), the HAdV E2A SCM mutant (left, lane 3; right, lane 4), and the HAdV E4orf3 mutant (left, lane 4; right, lane 5) at a multiplicity of infection of 20 FFU/cell, before harvesting cells 24 h after infection and preparing total-cell lysates. After immunoprecipitation by PML using polyclonal rabbit Ab raised against PML protein (catalog number NB100-59787) (anti-PML), proteins were subjected to immunoblot analyses. Input levels of total-cell lysates and coprecipitated proteins were detected using mAb B6-8 (anti-E2A), pAb NB100-59787 (anti-PML), mAb AC-15 (anti-β-actin), and E4orf3 antibody (6A11). Molecular weights in kilodaltons are indicated on the left, and relevant proteins are indicated on the right. (D) Densitometric analyses of E2A coimmunoprecipitated with PML in panel C (right, lanes 3 to 5), quantified with ImageJ (version 1.45s) software, normalized to the respective levels of immunoprecipitated PML in HAdV wt-, HAdV E2A SCM mutant-, and HAdV E4orf3 mutant-infected cells. Average values and standard deviations represented on the bar plot were calculated based on data from three independent experiments.

SUMOylation is involved in regulating the protein-protein interactions of its substrates in cells (65). PML is the scaffold protein of PML-NBs. PML preferentially recruits SUMOylated proteins through interactions of SUMO with its SUMO interaction motif (SIM) (6). Thus, we investigated whether the SUMOylation of E2A affects PML binding independently of any other viral component. Immunoprecipitation of cells transfected with the E2A-encoding plasmid pE2A-wt or pE2A-SCM showed that E2A SUMO conjugation does not influence PML binding without the viral background (Fig. 3A, right, lanes 3 and 4, and Fig. 3B).

During HAdV infection, PML bodies are reported to reorganize into PML tracks due to viral E4orf3 expression (31–36). To investigate the influence of E2A SUMOylation on E2A/PML binding and to ascertain any difference in the E2A/PML binding capacity due to a loss of PML-NB integrity during HAdV infection, we further performed immunoprecipitation of PML from cells infected with HAdV wt, HAdV E2A SCM, or HAdV ΔE4orf3. Subsequent detection of E2A revealed that E2A interaction with PML during infection depends on E2A SUMO PTM (Fig. 3C, right, lanes 3 and 4, and Fig. 3D). Compared to wt infection, E2A/PML binding was 71.8% lower when E2A SUMOylation was reduced by the triple-SCM mutation (Fig. 3C and D). Additional investigation of a virus mutant lacking E4orf3 showed no effect of this viral factor on E2A/PML binding (Fig. 3C, right, lane 5, and Fig. 3D). In sum, these data suggest that E2A SUMOylation regulates E2A/PML interaction during HAdV infection.

### E2A SUMOylation regulates the formation and localization of virus-induced PML tracks next to RCs.

**FIG 4:**
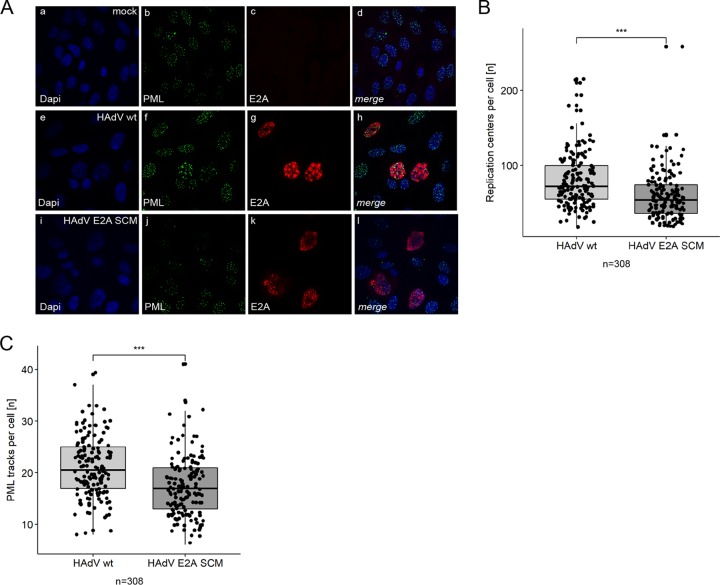
Abundances of HAdV RCs and PML-NBs depend on E2A SUMOylation. (A) HepaRG cells were infected with HAdV wt or HAdV E2A SCM at a multiplicity of infection of 50 FFU/cell, fixed with methanol at 24 h p.i. and double labeled with mAb B6-8 (anti-E2A) and pAb NB100-59787 (anti-PML). Primary Abs were detected with Alexa Fluor 647 (E2A) (red)- and Alexa Fluor 488 (PML) (green)-conjugated secondary Abs. Nuclear staining was performed using DAPI (4′,6-diamidino-2-phenylindole). Representative E2A (c, g, and k) and PML (b, f, and j) staining patterns of 308 cells analyzed are shown (50:50 ratio of HAdV wt/HAdV E2A SCM-infected cells). Overlays of single images (merge) are shown in panels d, h, and l. All cells were permeabilized, stained, and analyzed together to avoid differences in the staining intensity and extent of bleaching. (B and C) Using immunofluorescence microscopy, RCs and PML-NBs were counted per cell during infection with HAdV wt and HAdV E2A SCM using Volocity software. Numbers of replication centers (B) and PML-NBs (C) per cell were plotted in box plots. *P* values were calculated by employing a two-sided Mann-Whitney test. ***, *P* < 0.001.

**FIG 5:**
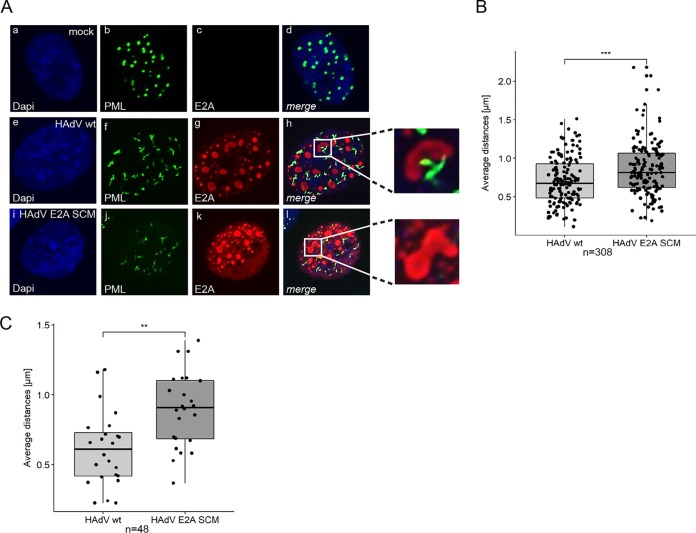
SUMO modification of E2A determines subcellular localization of PML tracks during HAdV infection. (A) HepaRG cells were infected with HAdV wt or HAdV E2A SCM at a multiplicity of infection of 50 FFU/cell, fixed with methanol at 24 h p.i., and double labeled with mAb B6-8 (anti-E2A) and pAb NB100-59787 (anti-PML). Primary Abs were detected with Alexa Fluor 647 (E2A) (red)- and Alexa Fluor 488 (PML) (green)-conjugated secondary Abs. DAPI was used for nuclear staining. Representative E2A (c, g, and k) and PML (b, f, and j) staining patterns of 308 cells analyzed are shown (50:50 ratio of HAdV wt/HAdV E2A SCM-infected cells). Overlays of single images (merge) are shown in panels d, h, and l. All cells were permeabilized, stained, and analyzed together to avoid differences in the staining intensity and extent of bleaching. (B) Distances between replication centers and PML tracks per cell were analyzed in 308 cells in 2D (50:50 ratio of HAdV wt/HAdV E2A SCM-infected cells), measured using Volocity software and represented in box plots. *P* values were calculated by employing a two-sided Mann-Whitney test. Bonferroni correction was used to adjust for multiple comparisons. (C) Cells were matched according to the numbers of PML-NBs and RCs, to avoid concentrations of each component confounding the correlation, before representing distances in box plots. *P* values were calculated by Mann-Whitney tests. Bonferroni correction was used to adjust for multiple comparisons. **, *P* < 0.01; ***, *P* < 0.001.

To investigate the effects of E2A SUMOylation on the organization of HAdV RCs and PML-NBs during infection, we performed immunofluorescence microscopy on cells infected with HAdV wt and HAdV E2A SCM to determine the morphology and abundance of these two structures (Fig. 4). The number of HAdV RCs per infected cell was 26.9% lower during HAdV E2A SCM infection than during HAdV wt infection (Fig. 4B). Moreover, the number of PML-NBs was significantly reduced by 17.7% compared to HAdV wt infection (Fig. 4C). Our results demonstrating an E2A SUMOylation-dependent E2A/PML interaction (Fig. 3C and D) suggested a novel interplay between E2A SUMOylation and PML track localization; supporting this, previous reports also showed that tracks are found in close proximity to E2A-marked HAdV RCs (31–33). Our immunofluorescence analysis of cells infected with HAdV wt confirmed such findings, substantiating the fact that virus-induced PML tracks are localized next to established HAdV RCs (Fig. 5A, panels f to h). However, in cells infected with HAdV E2A SCM, PML tracks were not associated with HAdV RCs but exhibited a predominantly random distribution in the cell nuclei (Fig. 5A, panels j to l). These findings imply that E2A SUMO PTM is involved in the so-far-unknown molecular mechanism underlying the adjacent recruitment of PML tracks to newly established RCs in HAdV-infected cells. To further validate our immunofluorescence data, we measured distances between HAdV RCs and PML tracks. Median distances of PML tracks from HAdV RCs were 21% longer during infection with HAdV E2A SCM (Fig. 5B).

Since E2A SUMOylation affected the numbers of HAdV RCs and PML tracks, we next determined how the distances between these two structures depend on their quantity. We observed a positive correlation between the increasing number of HAdV RCs and their distance from PML tracks during HAdV wt and HAdV E2A SCM infections (Fig. 5A; see also Fig. S1A in the supplemental material). In contrast, increasing numbers of PML tracks led to decreasing distances from HAdV RCs in wt and mutant virus infections (Fig. S1B). These correlations show that a meaningful comparison of average minimal distances between PML tracks and RCs (shown in Fig. 5B) requires the comparison of cells with similar amounts of these structures. Therefore, and to rule out the possibility that the concentrations of PML tracks and RCs confound the distance measurements, we matched cells for similar numbers of these structures. Our replotted data revealed a highly significant 48.8% increase in the median PML track distance from HAdV RCs during infection with the E2A SUMOylation-deficient virus HAdV E2A SCM (Fig. 5C). Taken together, our results show that E2A SUMO PTM represents a potential link between PML tracks and HAdV RCs, mediating their adjacent subcellular localization in infected cells.

10.1128/mBio.00049-20.1 FIG S1 The amounts of HAdV replication centers and PML tracks influence the distance between these two structures. HepaRG cells were infected with HAdV wt or HAdV E2A SCM at a multiplicity of 50 FFU/cell and analyzed by immunofluorescence microscopy. Numbers of HAdV RCs and PML tracks as well as distances between the two structures were determined using Volocity software. Correlations between numbers of replication centers (A) and PML tracks (B) and their distances per infected cell are represented in scatterplots. Correlation coefficients and statistical significances were computed using Spearman’s correlation. Download FIG S1, TIF file, 0.7 MB. Copyright © 2020 Stubbe et al. 2020 Stubbe et al. This content is distributed under the terms of the Creative Commons Attribution 4.0 International license.

### SUMOylation of E2A does not alter the DNA binding capacity of the viral protein.

Since we observed a significant correlation between E2A-containing HAdV RCs and PML track formation/localization being dependent on E2A SUMOylation, we further investigated the role of E2A SUMOylation during efficient HAdV replication.

**FIG 6:**
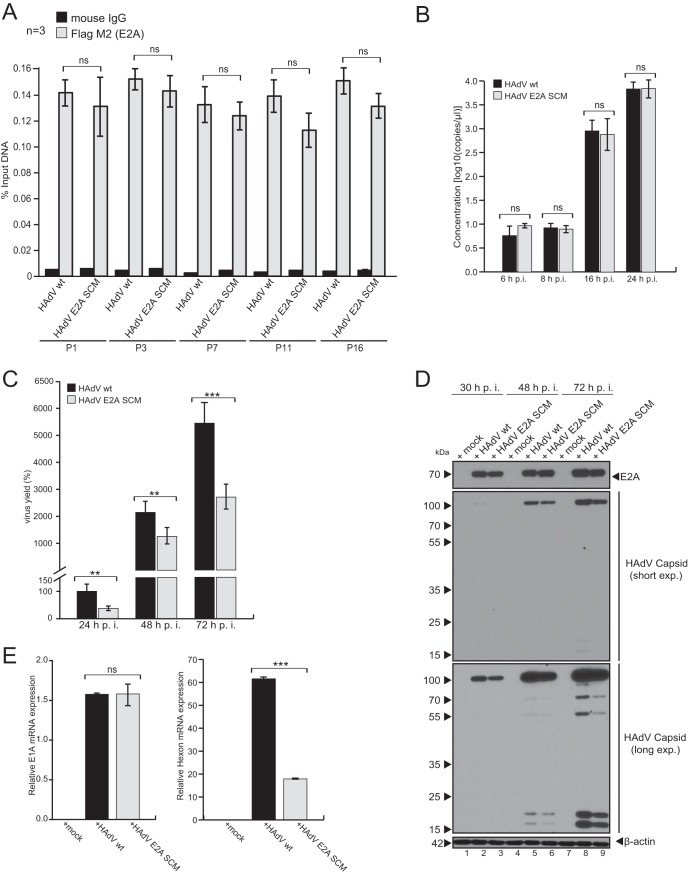
E2A SUMOylation promotes HAdV replication. (A) HepaRG cells were transfected with pFlag-E2A-wt or pFlag-E2A-SCM and infected 4 h later with the corresponding wt or mutant (E2A SCM) HAdV at a multiplicity of infection of 20 FFU/cell. Twenty-four hours after infection, cells were subjected to ChIP with an antibody to the Flag epitope tag or normal mouse IgG and primers specific for five indicator loci in the HAdV genome (P1, P3, P7, P11, and P16) (Table 2). The percentage of output versus input DNA was calculated, and values are given as the means ± standard deviations of data from three biological replicates. Student *t* tests were used to determine statistical significance. (B) HepaRG cells were infected with HAdV wt or the HAdV E2A SCM mutant at a multiplicity of infection of 20 FFU/cell and harvested at the indicated time points postinfection. Genomic DNA was isolated from the cells and amplified by RT-PCR with primer pairs specific for HAdV-C5 hexon (Table 2). The concentration of viral DNA was determined based on a standard curve with known concentrations of HAdV bacmid DNA. Statistics were calculated using Student’s *t* test. (C) Viral particles were harvested at 24, 48, and 72 h p.i., and virus yield was determined by quantitative capsid immunofluorescence staining in 2E2 cells. Statistically significant differences were assessed using a two-sided Mann-Whitney test. ns, not significant. (D) Cells were harvested at 30, 48, and 72 h p.i. Proteins from total-cell extracts were separated by SDS-PAGE and subjected to immunoblotting using mAbs AC-15 (anti-β-actin) and B6-8 (anti-E2A). Anti HAdV-C5 rabbit polyclonal serum L133 was used for the detection of capsid proteins. Molecular weights in kilodaltons are indicated on the left, and relevant proteins are indicated on the right. (E) Cells were harvested at 30 h p.i., and total RNA was extracted, reverse transcribed, and quantified by RT-PCR using primers specific for HAdV E1A (left plot) and hexon (right plot). The data were normalized to the respective GAPDH mRNA levels. Statistics were calculated using Student’s *t* test. **, *P* < 0.01; ***, *P* < 0.001; ns, not significant.

To determine whether E2A SUMO conjugation affects E2A binding to the viral genome, we performed chromatin immunoprecipitation (ChIP) assays with cells transfected with the pFlag-E2A-wt and pFlag-E2A-SCM constructs and subsequently infected with HAdV wt and HAdV E2A SCM, respectively. Sheared chromatin was subjected to immunoprecipitation using mouse anti-Flag M2 antibody (catalog number F3165; Sigma-Aldrich) or normal mouse IgG (catalog number I5381; Sigma-Aldrich) at 24 h postinfection (p.i.). A set of loci within the viral genome (P1, P3, P7, P11, and P16) (see Table 2) was selected to investigate E2A binding across the HAdV genome. Our results, gained by calculating the percentage of output versus input DNA, indicate that the distribution of E2A was remarkably uniform across all viral regions investigated, independent of E2A SUMOylation (Fig. 6A). These data are consistent with the results observed when we monitored DNA synthesis in E2A wt versus E2A SCM virus-infected cells (Fig. 6B), where we detected no difference between the samples for each independent time point postinfection. These data suggest that SUMOylation of the viral factor does not impair E2A functions in viral DNA binding and replication.

### SUMOylation of E2A is beneficial for HAdV replication.

To investigate whether E2A SUMOylation affects the production of adenoviral progeny, virus growth was determined in HepaRG cells (Fig. 6C). Our results pointed to progeny production depending on E2A SUMOylation at all three time points examined postinfection. We detected 62% less viral progeny production 24 h after infection with HAdV E2A SCM. Compared to HAdV wt infection, the level of production of viral progeny was lower by 41% after 48 h and reached 50% after 72 h (Fig. 6C).

To examine the influence of SUMO PTM on E2A stability, we monitored protein levels of E2A during infection with HAdV wt and HAdV E2A SCM. Even though our data showed that viral progeny production depends on E2A SUMOylation (Fig. 6C), E2A SCM mutations did not affect E2A protein expression during HAdV E2A SCM infection (Fig. 6D). Consistent with the virus yield data, we observed lower late protein expression levels during E2A SCM mutant virus infection than during HAdV wt infection at 30, 48, and 72 h postinfection (Fig. 6D). Similar results were obtained by monitoring viral hexon (Fig. 6E, left) mRNA synthesis in infected cells, while E1A mRNA synthesis was not affected by E2A SCM mutations (Fig. 6E, right).

To further strengthen these observations, adenoviral progeny production, late protein expression, and late mRNA synthesis were determined in H1299 cells (Fig. S2). Our data showed 26% less viral progeny production 48 h after infection with HAdV E2A SCM and 31% less progeny production after 72 h than for HAdV wt infection (Fig. S2A). The lower level of late protein expression during E2A SCM mutant infection than during HAdV wt infection at 24, 48, and 72 h postinfection was consistent with the virus yield data (Fig. S2B). The level of hexon mRNA synthesis in cells infected with HAdV E2A SCM was 70% lower than for HAdV wt infection (Fig. S2C).

10.1128/mBio.00049-20.2 FIG S2 E2A SUMOylation promotes HAdV replication. (A) H1299 cells were infected with HAdV wt or the HAdV E2A SCM mutant at a multiplicity of 5 FFU/cell and harvested at 24, 48, and 72 h postinfection (p.i.). The virus yield was determined by quantitative capsid immunofluorescence staining in 2E2 cells. Statistically significant differences were assessed using Student’s *t* test. (B) H1299 cells were harvested at 24, 48, and 72 h p.i. Proteins from total-cell extracts were separated by SDS-PAGE and subjected to immunoblotting using mAb AC-15 (anti-β-actin) and anti-HAdV-C5 rabbit polyclonal serum L133 for the detection of capsid proteins. Molecular weights in kilodaltons are indicated on the left, and relevant proteins are indicated on the right. (C) Cells were harvested at 30 h p.i., and total RNA was extracted, reverse transcribed, and quantified by RT-PCR using primers specific for HAdV hexon. The data were normalized to the respective GAPDH mRNA levels. Statistics were calculated using Student’s t test. Download FIG S2, TIF file, 1.1 MB. Copyright © 2020 Stubbe et al. 2020 Stubbe et al. This content is distributed under the terms of the Creative Commons Attribution 4.0 International license.

### E2A SUMOylation does not affect Sp100A sequestration into PML tracks.

**FIG 7:**
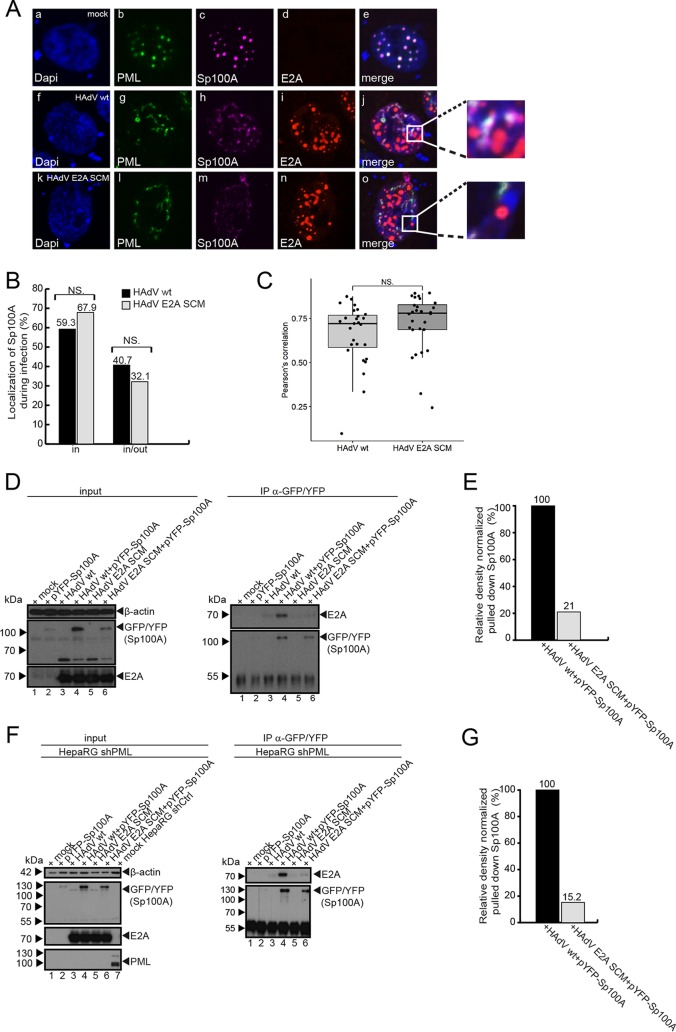
E2A SUMOylation promotes Sp100A-E2A interaction. (A) HepaRG cells were transfected with 3 μg of pYFP-Sp100A and superinfected with HAdV wt or the HAdV E2A SCM mutant at a multiplicity of infection of 50 FFU/cell at 4 h posttransfection. The cells were fixed with 4% paraformaldehyde (PFA) 30 h after infection and double labeled with mAb rb (anti-E2A) and pAb m (anti-PML). Sp100A was not labeled but was detected via YFP fluorescence (Sp100A) (magenta). Primary Abs were detected with Alexa Fluor 568 (E2A) (red)- and Alexa Fluor 647 (PML) (green)-conjugated secondary Abs. DAPI was used to stain nuclei. Anti-PML (green) (b, g, and l) and anti-YFP (c, h, and m) staining patterns are representative of results from at least 50 cells analyzed. Overlays of the single images (merge) are shown in panels e, j, and o. (B) Cells were categorized according to intracellular YFP-Sp100A localization in PML-NBs, and the counting data are visualized in a bar plot. Statistical significance was calculated using a Fisher exact test (*P* > 0.05). (C) Volocity software was used to determine Pearson’s correlation coefficient for YFP-Sp100A signals and PML stained by PML mouse Ab (anti-PML) in cells infected with HAdV wt or HAdV E2A SCM. For each cell, Pearson’s correlation was calculated for the colocalization of PML with YFP-Sp100A. The distributions of the respective correlation coefficients are displayed in a box plot. Values above 0.5 are considered colocalization. NS, not significant. (D) HepaRG cells were transfected with pYFP-Sp100A (lane 2) and superinfected with HAdV wt (lane 4) and the HAdV E2A SCM mutant (lane 6) at a multiplicity of infection of 50 FFU/cell. The cells were harvested at 24 h p.i., and whole-cell lysates were prepared. Immunoprecipitation analysis was performed using a polyclonal rabbit antibody against GFP/YFP, pAb260 (anti-GFP/YFP). Proteins were subjected to immunoblot analyses. Input levels of total-cell lysates and coprecipitated proteins were detected using mAb B6-8 (anti-E2A), pAb260 (anti-GFP/YFP), and mAb AC-15 (anti-β-actin). Molecular weights in kilodaltons are indicated on the left, and relevant proteins are indicated on the right. (E) Densitometric analysis of E2A interactions with Sp100A in panel D (lanes 4 and 6), quantified with ImageJ (version 1.45s) software, normalized to the respective levels of immunoprecipitated YFP-Sp100A. (F) HepaRG shPML cells were transfected with pYFP-Sp100A (lane 2) and superinfected with HAdV wt (lane 4) and the HAdV E2A SCM mutant (lane 6) at a multiplicity of infection of 50 FFU/cell. The cells were harvested at 24 h p.i., and whole-cell lysates were prepared. HepaRG shCtrl cells were used to verify PML depletion in HepaRG shPML cells (left, lane 7). Immunoprecipitation analysis was performed using a polyclonal rabbit antibody against GFP/YFP, pAb260 (anti-GFP/YFP). After immunoblotting, input levels of total-cell lysates and coprecipitated proteins were detected using mAb B6-8 (anti-E2A), pAb260 (anti-GFP/YFP), pAb NB100-59787 (anti-PML), and mAb AC-15 (anti-β-actin). Molecular weights in kilodaltons are indicated on the left, and relevant proteins are indicated on the right. (G) Densitometric analysis of E2A interaction with Sp100A in panel F (lanes 4 and 6), quantified with ImageJ (version 1.45s) software, normalized to the respective levels of immunoprecipitated YFP-Sp100A.

Sp100 is one of the main components in assembled PML-NBs and localizes within these dot-like structures. HAdV infection causes the reorganization of PML-NBs into PML tracks and the redistribution of Sp100 isoforms. We recently reported that Sp100 isoforms B, C, and HMG are sequestered into viral RCs, while Sp100A remains in PML tracks, benefiting the virus by amplifying HAdV gene expression (45). To assess the role of E2A SUMOylation in Sp100A positioning in PML-NBs, we transfected cells with pYFP-Sp100A prior to infection with HAdV wt and HAdV E2A SCM. Our immunofluorescence data confirmed previous observations that Sp100A localizes in PML tracks during HAdV wt infection (Fig. 7A, panels g and h, and Fig. S3, panels j, o, and t). Analysis of cells infected with HAdV E2A SCM revealed that Sp100A was localized in PML tracks randomly distributed in cell nuclei (Fig. 7A, panels l and m, and Fig. S3, panels y, d1, and i1). In comparison to HAdV wt infection, we did not observe any change in Sp100A localization (Fig. 7B). This is further supported by results showing no difference in Pearson’s correlations between Sp100A and PML (Fig. 7C).

10.1128/mBio.00049-20.3 FIG S3 E2A SUMOylation does not affect Sp100A localization. HepaRG cells were transfected with 3 μg of pYFP-Sp100A and superinfected with HAdV wt or the HAdV E2A SCM mutant at a multiplicity of 50 FFU/cell at 4 h posttransfection. The cells were fixed with 4% PFA 30 h after infection and double labeled with mAb rb (anti-E2A) and pAb m (anti-PML). Sp100A was not labeled but was detected via YFP fluorescence (magenta). Primary Abs were detected with Alexa 568 (E2A) (red)- and Alexa 647 (PML) (green)-conjugated secondary Abs. DAPI was used to stain nuclei. Anti-PML (green) (panels b, g, l, q, v, a1, and f1) and anti-YFP (magenta) (panels c, h, m, r, w, b1, and g1) staining patterns are representative of results for at least 50 cells analyzed. Overlays of the single images (merge) are shown in panels e, j, o, t, y, d1, and i1. Download FIG S3, TIF file, 1.2 MB. Copyright © 2020 Stubbe et al. 2020 Stubbe et al. This content is distributed under the terms of the Creative Commons Attribution 4.0 International license.

### E2A SUMOylation promotes interaction with the HAdV transcriptional activator Sp100A localized in PML tracks.

Sp100A is associated with PML tracks during wt HAdV infection (45), independent of the E2A SUMO status. After human cells were transfected with pYFP-Sp100A and superinfected with wt HAdV or the E2A SCM virus mutant, immunoprecipitation of yellow fluorescent protein (YFP) and staining for E2A revealed an interaction between Sp100A and E2A (Fig. 7D, right, lane 4, and Fig. 7E). A smaller amount of E2A protein was detected when Sp100A interactions were monitored in the presence of the SCM mutant variant, confirming an E2A SUMO-dependent interaction between the viral factor and the host transcription factor (Fig. 7D, right, lane 6, and Fig. 7E). We note that during wt HAdV infection, Sp100A protein levels were higher (Fig. 7D, left, lane 4) than those during SCM mutant virus infections (Fig. 7D, left, lane 6).

To next determine whether PML mediates Sp100A/E2A interactions, we transfected PML-depleted cells with pYFP-Sp100A and subsequently infected the cells with wt HAdV or the E2A SCM mutant virus. Immunoprecipitation of YFP and staining for E2A revealed a PML-independent interaction between Sp100A and E2A (Fig. 7F, right, lane 4). Taken together, Sp100A/E2A interactions (Fig. 7F, right, lane 6, and Fig. 7G) and Sp100A protein levels (Fig. 7F, left, lane 6) are also regulated by E2A SUMOylation in the absence of PML; this result highlights a novel role for the viral E2A factor regulated by its own SUMO PTM to promote efficient HAdV infection.

### E2A SUMOylation-dependent localization of PML tracks next to viral replication centers promotes HAdV infection.

**FIG 8:**
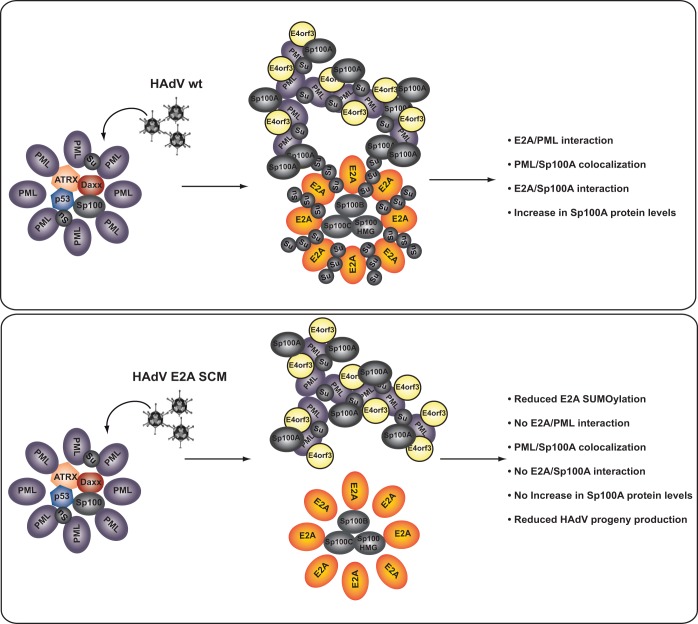
Model of E2A SUMO modification-mediated localization of PML tracks next to viral replication centers during infection. Shown is a schematic representation illustrating a proposed model linking E2A SUMOylation during infection with PML-NB subcellular localization. The model shows the influence of E2A SUMO PTM on PML-NB localization adjacent to HAdV RCs by E2A SUMOylation-dependent E2A/PML and E2A/Sp100A interactions. E2A SCM mutations prevent the virus from using beneficial components from PML-NBs, leading to defects in virus gene expression and progeny production.

Our data showed that E2A is a novel target of the host SUMOylation machinery (Fig. 1, 2, and 8, top). SUMOylated E2A interacts with PML (Fig. 3C and Fig. 8, top), and PML-NBs localize adjacent to HAdV RCs in HAdV wt infection (31–33, 36) (Fig. 8, top). Infection with HAdV wt results in higher levels of Sp100A protein (Fig. 7D and F and Fig. 8, top) and PML-independent interactions between E2A and Sp100A (Fig. 7D and F and Fig. 8, top). In the absence of E2A SUMOylation, there are fewer E2A/PML (Fig. 3C and D and Fig. 8, bottom) and E2A/Sp100A (Fig. 7D to G and Fig. 8, bottom) interactions and increases in Sp100A levels are lower than in wt infection (Fig. 7D and F and Fig. 8, bottom). E2A SCM mutations led to larger distances between RCs and PML tracks than in HAdV wt infection (Fig. 5A to C and Fig. 8, bottom). Based on these observations, we hypothesize that HAdV E2M SCM is no longer capable of using infection-promoting components of PML tracks (30, 45), which leads to less HAdV progeny production than in HAdV wt infection (Fig. 6C and Fig. 8, bottom).

## DISCUSSION

HAdVs (human adenoviruses) exploit the host cell’s SUMOylation machinery, not only to benefit from the transcription-activating properties of PML-NB constituents but also to simultaneously counteract PML-NBs’ antiviral activity. PML-NBs are necessary for establishing efficient replication and transcription centers of different DNA viruses. In particular, the transcription of HCMV (human cytomegalovirus) and HSV-1 (herpes simplex virus 1) genomes also occurs at PML-NBs (33, 34, 71). Moreover, the replication of HSV-1 is supported by the recruitment of several cellular DNA repair factors to viral replication compartments (72). The transcription and replication of SV40 (simian virus 40) and HPyVs (human polyomaviruses) also take place in proximity to PML-NBs (73, 74).

In the case of HAdVs, upon entering a cell, the viral DNA rapidly localizes adjacent to PML-NBs by so-far-unknown mechanisms. During the early phase, PML-NBs are reorganized into PML tracks by the HAdV early protein E4orf3 (31, 75). HAdV RCs, defined by the HAdV DNA binding protein E2A, are formed in close proximity to PML tracks and become designated sites of active viral transcription (31, 56). Our findings reveal that E2A is a novel interaction partner of PML and Sp100A during infection, indicating that E2A plays a role in HAdV manipulation of PML-NB functions.

Based on our experiments, we conclude that the interaction between E2A and PML is independent of E2A SUMOylation alone. We conclude that E4orf3 or track formation is not involved in this process, as it has been reported that even when PML is not redistributed to tracks, as in the case of an E4orf3-depleted virus, RCs still form in proximity to PML-NBs (33), and we still observe E2A/PML interactions in the absence of E4orf3 during infection. We hypothesize that during infection, most E2A will be bound to HAdV DNA, masking its DNA binding domain and potentially also other surfaces that might mediate direct interactions with PML, so only SUMO-SIM interactions are seen. In addition, another reason would be the organization of E2A in RCs together with different SUMO proteins in infection. It was recently reported that during infection, SUMO-1 localizes outside RCs, whereas SUMO-2 and SUMO-3 are mainly detected inside RCs (76). This RC/infection-mediated change of the localization of SUMO proteins involved in the E2A/PML interaction could explain a change in the binding capacity.

HAdVs are tightly connected to the host SUMOylation system, using it to manipulate host factors to establish productive infection (43, 44, 77–82). During infection with E2A SCM virus with reduced E2A SUMOylation, the localization of PML tracks was displaced from the HAdV RCs. This might prevent the virus from using beneficial PML components retained in virus-induced tracks by abolishing the contact of PML tracks with newly synthesized HAdV mRNA. As PML tracks represent sites of active viral transcription (31, 76), it is not surprising that infection with the E2A SCM mutant virus resulted in a reduced replication efficacy. Many transcriptional repressor proteins, such as Daxx, p53, or the Sp100 isoforms B, C, and HMG, localize within PML-NBs and contribute to the antiviral properties of these compartments (45–47, 83, 84). To counteract this function of PML-NBs, HAdVs have acquired various mechanisms. First, the integrity of PML-NBs is disrupted by the early HAdV protein E4orf3 (31, 75). Consequently, repressive components from PML-NBs become accessible to HAdV proteins involved in inhibiting the cellular antiviral response. One of these viral factors is E1B-55K localized in virus-induced PML tracks and involved in the assembly of the HAdV E3 ubiquitin ligase complex that proteasomally degrades repressive cellular factors (31, 41, 43, 44, 85–91) or the recruitment of the SUMO-dependent ubiquitin ligase RNF4 (92). An additional mechanism employed by HAdV to counteract host antiviral strategies in order to replicate is the active displacement of inhibitory host factors, such as Sp100 isoform B, into the interior of HAdV RCs (45). Here, we show that Sp100A, an Sp100 isoform beneficial for HAdV infection (45), is efficiently bound by SUMOylated E2A. We speculate here that the Sp100A-mediated activation of HAdV gene expression is even promoted by the increase in the Sp100A quantity during wt HAdV infection. We did not detect this in cells infected with the E2A SUMOylation-deficient virus; thus, SUMOylation might be involved.

Ubc9 represents the main SUMO-conjugating enzyme also involved in Sp100 SUMOylation (51). Thus, the inability of the E2A SCM to interact with Ubc9 might prevent an HAdV-mediated decrease in Sp100A SUMO moieties and thus block the transcription-activating properties (93). The loss of Sp100A protein levels and faulty PML track/RC cooperation in E2A SCM infection might in sum prevent the virus from exploiting beneficial Sp100A capacity. Based on the fact that our data revealed PML-independent and E2A SUMOylation-dependent binding between Sp100A and E2A, we are tempted to speculate that Sp100A is the unknown factor bridging RCs to PML tracks.

Our observations provide insight into novel HAdV cross talk with the host SUMOylation machinery to exploit the beneficial components of PML-NBs. E2A SUMOylation is beneficial during HAdV infection; this DNA virus determinant regulates binding to PML and Sp100A during infection, thus promoting PML track localization next to HAdV RCs as a prerequisite for efficient viral infection and a potential target structure for future HAdV intervention strategies.

## MATERIALS AND METHODS

### Cell culture.

HepaRG cells (96, 103) (Thermo Scientific), HepaRG cells stably expressing His/hemagglutinin (HA)–SUMO-2 (kind gift of Roger Everett, University of Glasgow), HepaRG shPML cells (44, 95), H1299 cells (94) (ATCC CRL-5803), HEK-293 cells (Sigma-Aldrich Inc.), 2E2 cells (97), and HeLa cells stably expressing 6×His–SUMO-2 (98) were grown in Dulbecco’s modified Eagle’s medium (DMEM) supplemented with 10% fetal calf serum (FCS), 100 U of penicillin, and 100 μg of streptomycin per ml at 37°C in an atmosphere with 5% CO_2_. For the propagation of HepaRG cells, medium was additionally supplemented with 5 μg/ml of bovine insulin and 0.5 μM hydrocortisone. HeLa/HepaRG 6×His-SUMO/HepaRG shPML cell lines were maintained under 2 μM puromycin selection. 2E2 cells were maintained under 90 μg/μl hygromycin B and 250 μg/μl of G-418. Helper function was induced with 1 μg/ml of doxycycline in the above-described media. All cell lines were regularly tested for mycoplasma contamination.

### Plasmids, mutagenesis, and transient transfection.

Mutations were introduced into the E2A gene by site-directed mutagenesis using oligonucleotides (Table 1). pCMX3b-Flag E2A (pFlag-E2A-wt) and pCMX3b-Flag E2A (pFlag-E2A) variants were used to transfect HepaRG SUMO-2 cells for Ni-NTA analysis. pCMX3b-E2A (pE2A-wt) and pCMX3b-E2AK94/132/202R (pE2A-SCM), as well as pGFP-E2A (pGFP-E2A-wt) and pGFP-E2AK94/132/202R (pGFP-E2A-SCM) cloned into a pEGFP-C1 vector (KpnI and BamHI), were used for transfections for immunoprecipitation analyses in H1299 and HepaRG cells.

**TABLE 1:** Cloning primers used in this study

Primer	Sequence (5′→3′)
Sp100A fwd	ATAAAGCTTATATGGCAGGTGGGGGCGGC
Sp100A rev	TATCCCGGGCATCTTCTTTACCTGACCCTCTTC
E2A K94R fwd	GAAGCGCCCTTCTCCCAGGCCCGAGCGCCCG
E2A K94R rev	CGGGCGCTCGGGCCTGGGAGAAGGGCGCTTC
E2A K202R fwd	GTGGATAACGATCTAAGGGCGAACTTCAAACTAC
E2A K202R rev	GTAGTTTGAAGTTCGCCCTTAGATCGTTATCCAC
E2A K132R fwd	GCTAATCAAGCATGGCAGAGGAGGTAAGCGCACAG
E2A K132R rev	CTGTGCGCTTACCTCCTCTGCCATGCTTGATTAGC

The N-terminally YFP-tagged human Sp100 isoform A construct was generated using specific oligonucleotides (Table 1). The recently described (45, 50) N-terminally Flag-tagged construct pFlag-Sp100A was used as the template. Sp100A was inserted into the pEYFP vector (Clontech) using the restriction enzymes HindIII and SmaI. For yeast experiments, cDNAs of selected open reading frames (ORFs) (UBE2E1, UBC9, SUMO-1, PIAS4, OTUB1, SUMO-2, and full-length adenoviral E2A) were amplified via PCR and cloned into the pGAD-C1 and pGBD-C1 plasmids using the restriction enzymes EcoRI and BamHI. For transient transfections, a mixture of DNA and 25-kDa linear polyethylenimine (Polysciences) was applied to subconfluent cells as previously described (44).

### Viruses.

In this work, H5*pg*4100 was used as the wild-type HAdV-C5 parental strain (HAdV wt) (44). The HAdV E2A SCM mutant was generated as described previously (99). This virus carries point mutations in the E2A gene leading to the exchange of amino acids at positions 94, 132, and 202 from lysine to arginine, disrupting the SCM in E2A. Viruses were propagated in H1299 cells and titrated in 2E2 cells (99) with an inducible E2A helper function as described previously (97). The virus yield was determined by quantitative HAdV capsid immunofluorescence staining in 2E2 cells. Viral DNA replication was monitored by quantitative PCR (qPCR) using hexon-specific primers (Table 2). Cells were lysed with radioimmunoprecipitation assay (RIPA) buffer as previously described (100), and total DNA was isolated by proteinase K digestion. Samples were diluted 1:100 in nucleic acid-free water (Promega), and 2 μl per sample was mixed with 12.5 μl of Power SYBR green PCR master mix (Roche) and a 10 μM concentration of the appropriate primer pairs in a total volume of 24 μl. The concentration of viral DNA was calculated based on a standard curve obtained by quantitative PCR analysis for known concentrations of HAdV bacmid DNA.

**TABLE 2:** qPCR primers used in this study

Primer	Sequence (5′→3′)	Target
HAdV P1 fwd	CTTGCATGGCGTGTTAAATG	HAdV positions 1635–1654
HAdV P1 rev	GCCTCCATGAGGTCAGATGT	HAdV positions 1702–1721
HAdV P3 fwd	GGTGCACTTGGGAAATTTGT	HAdV positions 4508–4527
HAdV P3 rev	TATGGACGAATGCATGGAAA	HAdV positions 4588–4607
HAdV P7 fwd	TGACGAGGACGATGAGTACG	HAdV positions 12255–12274
HAdV P7 rev	GTTGCGTCTTGCATCATCTG	HAdV positions 12315–12334
HAdV P11 fwd	CTGTGGGTGATAACCGTGTG	HAdV positions 19101–19120
HAdV P11 rev	TAAAAGTAGGGCCCCTGTCC	HAdV positions 19162–19181
HAdV P16 fwd	AACGCCATAGTTGCTTGCTT	HAdV positions 26588–26607
HAdV P16 rev	ACGCCGTGATGGTAGAGAAG	HAdV positions 26648–26667
GAPDH fwd	CATCCTGGGCTACACTGA	
GAPDH fwd	TTGACAAAGTGGTCGTTG	
E1A fwd	GTGCCCCATTAACCAGTTG	
E1A rev	GGCGTTTACAGCTCAAGTCC	
Hexon fwd	CGCTGGACATGACTTTTGAG	
Hexon rev	GAACGGTGTGCGCAGGTA	

### Yeast two-hybrid assay.

Competent Saccharomyces cerevisiae cells were prepared as previously described (80). The yeast two-hybrid expression plasmid pGAD-C1 or pGBD-C1 was used to fuse the GAL4 transcription factor activation domain (AD) or binding domain (BD) to the proteins of interest. AD and BD plasmids contained Leu and Trp as markers, respectively. Cotransformation of these plasmids in PJ69-7A cells was performed according to a standard protocol (80) and plated onto selection medium lacking Leu and Trp (containing His) to monitor successful transformation and spotted onto selection medium lacking His, Leu, and Trp to monitor protein-protein interactions.

### Protein analysis and antibodies.

Cells for protein analysis were resuspended in RIPA buffer as previously described (100). After 30 min on ice, the lysates were sonicated, and the insoluble debris was pelleted at 11,000 rpm at 4°C. For immunoprecipitation, samples were precleared by adding pansorbin cells (Millipore Calbiochem). Protein A-Sepharose beads (3 mg/sample) were coupled with 0.25 μl of polyclonal rabbit antibody raised against PML protein (anti-PML) or with 0.25 μl of polyclonal rabbit anti-GFP Ab for 1 h at 4°C. Antibody-coupled protein A-Sepharose was added to precleared samples and rotated for 1 h at 4°C. Proteins bound to antibody-coupled protein A-Sepharose were centrifuged, washed twice, boiled for 5 min at 95°C in 2× Laemmli buffer, and subsequently analyzed by Western blotting as previously described (77). Denaturing purification and analysis of SUMO conjugates were performed as described previously (77). To detect ubiquitin, PTM cells were transfected with His-ubiquitin (His-Ubi) constructs, cell harvesting was performed at 4°C, and denaturing purification was performed as described previously (45). Primary Abs specific for HAdV proteins used in this study included E2A mouse monoclonal antibody (mAb) B6-8 (101), E4orf3 antibody (clone 6A11), and polyclonal rabbit serum against HAdV-C5 capsid L133 (78). Primary Abs specific for cellular proteins included monoclonal mouse Ab AC-15 against β-actin (catalog number A5441; Sigma-Aldrich), monoclonal mouse Ab against Ubc9 (clone C-12; Santa Cruz Biotechnology), polyclonal rabbit anti-GFP/GFP Ab (catalog number ab290; Abcam), monoclonal mouse Ab against the 6×His epitope (catalog number 631213; Clontech), and polyclonal rabbit Ab (catalog number NB100-59787; Novus Biologicals) and monoclonal mouse Ab (catalog number sc-966; Santa Cruz Biotechnology), both raised against the PML protein. Secondary Abs conjugated to HRP (horseradish peroxidase) used to detect proteins by immunoblotting were anti-rabbit IgG and anti-mouse IgG (Jackson/Dianova).

### Quantitative real-time PCR analysis.

Subconfluent cells were infected with HAdV and harvested at 30 h p.i. Total RNA was isolated with TRIzol reagent (Invitrogen) as described by the manufacturer. The amount of total RNA was measured, and 1 μg of RNA was reverse transcribed using a reverse transcription kit from Promega, including an anchored oligo(dT)_15_ primer specific for poly(A)^+^ RNA. Quantitative real-time PCR (qRT-PCR) was performed with a first-strand method in 0.5-ml reaction tubes containing a 1/100 dilution of the cDNA template, 10 pmol/ml of each synthetic oligonucleotide primer, and 12.5 ml/sample Power SYBR green PCR master mix (Roche). The PCR conditions were as follows: 10 min at 95°C and 55 cycles of 30 s at 95°C, 30 s at 55 to 62°C, and 30 s at 72°C. The average threshold cycle (*C_T_*) value was determined from triplicate reactions, and levels of viral mRNA relative to cellular glyceraldehyde-3-phosphate dehydrogenase (GAPDH) mRNA were calculated as described recently (71). The identities of the products obtained were confirmed by melting-curve analysis.

### Chromatin immunoprecipitation assay.

HepaRG cells were transfected with pFlag E2A-wt or pFlag E2A SCM constructs and after 4 h infected with the corresponding HAdV. The cells were cross-linked with 1% methanol-free formaldehyde (Thermo Fisher Scientific) for 10 min at room temperature, 24 h after infection. Glycine was added to a final concentration of 125 mM to stop the cross-linking reaction, and the samples were incubated for another 5 min at room temperature. Cells were washed twice with ice-cold phosphate-buffered saline (PBS), harvested, and lysed in SDS buffer (50 mM Tris-HCl [pH 8.1], 10 mM EDTA, 1% SDS). Samples were incubated on ice for 10 min, sonicated for 15 min in a Diagenode Bioruptor Pico (30-s on-off intervals), and cleared by centrifugation two times for 10 min at 16,000 × *g* at 4°C. For chromatin immunoprecipitation (ChIP), sheared chromatin from 1.5 × 10^6^ cells in 50 μl (Flag/E2A-ChIP) was combined with 9 volumes of ChIP dilution buffer (16.7 mM Tris-HCl [pH 8.1], 167 mM NaCl, 1.2 mM EDTA, 1.1% Triton X-100, 0.01% SDS) and subjected to immunoprecipitation for 16 h at 4°C with gentle rotation using 5 μg mouse anti-Flag M2 antibody (catalog number F3165; Sigma-Aldrich), or 5 μg normal mouse IgG (catalog number I5381; Sigma-Aldrich). Samples were centrifuged for 10 min at 20,000 × *g* at 4°C to remove any precipitated material, and the supernatants were combined with 20 μl Magna ChIP protein G (E2A/Flag-ChIP) magnetic beads (Millipore) and incubated for 2 h at 4°C with gentle rotation. Immune complexes were washed consecutively with 1 ml each of low-salt buffer (20 mM Tris-HCl [pH 8.1], 150 mM NaCl, 2 mM EDTA, 1% Triton X-100, 0.1% SDS), high-salt buffer (20 mM Tris [pH 8.1], 0.5 M NaCl, 2 mM EDTA, 1% Triton X-100, 0.1% SDS), and lithium chloride buffer (10 mM Tris [pH 8.1], 0.25 M LiCl, 1 mM EDTA, 1% IGEPAL CA-630, 1% deoxycholic acid) and twice with TE (10 mM Tris-HCl [pH 8.0], 1 mM EDTA) buffer. Elution of the chromatin-antibody complexes was carried out by incubation with 150 μl freshly prepared elution buffer (100 mM NaHCO_3_, 1% SDS) containing 1.5 μl proteinase K (Roche) at 62°C for 2 h, followed by a 10-min incubation step at 95°C. DNA was purified using the MinElute PCR purification kit from Qiagen according to the manufacturer’s instructions. DNA was eluted twice with 20 μl buffer EB, and 2 μl of the DNA solution was used as the template DNA for qPCR with the primers listed in Table 2.

### Indirect immunofluorescence.

Cells grown on glass coverslips were transfected and/or infected. Immunofluorescence experiments were performed using previously described protocols (78). Digital images were acquired using a Nikon TiE microscope equipped with the Perkin-Elmer UltraView Vox system. The colocalization of PML and Sp100A was analyzed by determining Pearson’s correlation coefficient. Average minimal distances between PML tracks and RCs were measured in two dimensions (2D) in 154 cells infected with HAdV wt and in 154 cells infected with the HAdV E2A SCM mutant. Pictures were analyzed with Volocity 6.2.1 software (Perkin-Elmer). Images were cropped using Adobe Photoshop CS6 and assembled with Adobe Illustrator CS6.

### Statistical analysis.

Testing for statistically significant differences in medians was performed using a two-sided Mann-Whitney test. For normally distributed data, differences in means were tested for statistical significance using Student’s *t* test. Fisher’s exact test was employed to test for differences in proportions of counting data. Bonferroni correction was used to adjust *P* values for multiple comparisons. Spearman’s correlation was used to assess relationships between variables. Correlations were tested for statistical significance using algorithm AS 89 (102). All statistical analyses were carried out using the R language and environment for statistical computing.
