# Supplementary figures and images for: Viral DNA Binding Protein SUMOylation Promotes PML Nuclear Body Localization Next to Viral Replication Centers
Source: mBio. 2020 Mar 17;11(2):e00049-20. doi: 10.1128/mBio.00049-20 (PMC7078464; doi:10.1128/mBio.00049-20)

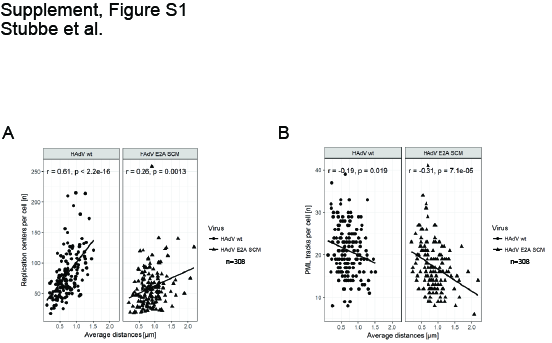

Supplement: FIG S1 [file mBio.00049-20-sf001.tif]

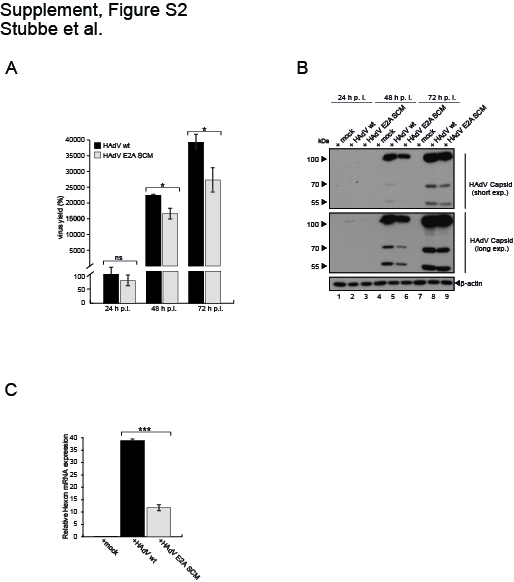

Supplement: FIG S2 [file mBio.00049-20-sf002.tif]

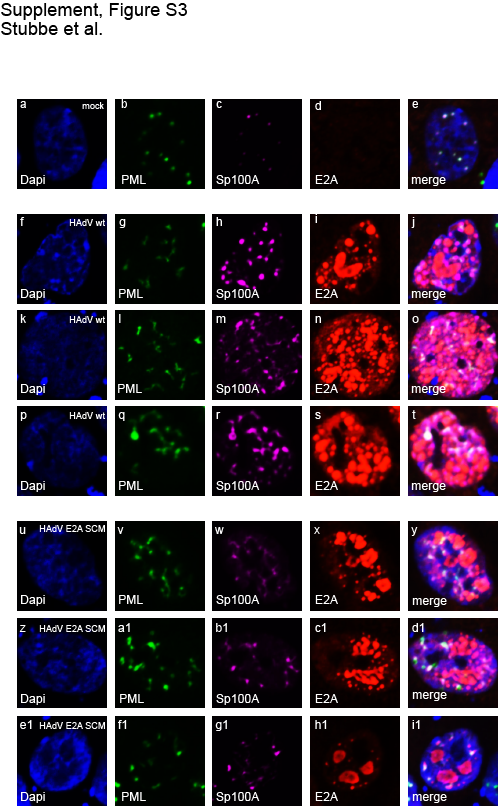

Supplement: FIG S3 [file mBio.00049-20-sf003.tif]
